# Seasonal Variations in Triple Oxygen Isotope Ratios of Precipitation in the Western and Central United States

**DOI:** 10.1029/2022pa004458

**Published:** 2023-03-13

**Authors:** P. G. Aron, S. Li, J. R. Brooks, J. M. Welker, N. E. Levin

**Affiliations:** 1 Department of Earth and Environmental Sciences, University of Michigan, Ann Arbor, MI, USA; 2 Now at Hazen and Sawyer, Baltimore, MD, USA; 3 School of Earth and Space Sciences, Institute of Geochemistry, Peking University, Beijing, China; 4 Pacific Ecological Systems Division, Center for Public Health and Environmental Assessment, Office of Research and Development, U.S. Environmental Protection Agency, Corvallis, OR, USA; 5 Department of Biological Sciences, University of Alaska, Anchorage, AK, USA; 6 Ecology and Genetics Research Unit, University of Oulu, Oulu, Finland; 7 University of the Arctic (UArctic), Rovaniemi, Finland

## Abstract

Triple oxygen isotope ratios $\left(\Delta^{\prime 17}O\right)$ offer new opportunities to improve reconstructions of past climate by quantifying evaporation, relative humidity, and diagenesis in geologic archives. However, the utility of $\Delta^{\prime 17}O$ in paleoclimate applications is hampered by a limited understanding of how precipitation $\Delta^{\prime 7}O$ values vary across time and space. To improve applications of $\Delta^{\prime 17}O$, we present $\delta^{18}O$, *d*-excess, and $\Delta^{\prime 17}O$ data from 26 precipitation sites in the western and central United States and three streams from the Willamette River Basin in western Oregon. In this data set, we find that precipitation $\Delta^{\prime 17}O$ tracks evaporation but appears insensitive to many controls that govern variation in $\delta^{18}O$, including Rayleigh distillation, elevation, latitude, longitude, and local precipitation amount. Seasonality has a large effect on $\Delta^{\prime 17}O$ variation in the data set and we observe higher seasonally amount-weighted average precipitation $\Delta^{\prime 17}O$ values in the winter (40 ± 15 per meg [± standard deviation]) than in the summer (18 ± 18 per meg). This seasonal precipitation $\Delta^{\prime 17}O$ variability likely arises from a combination of sub-cloud evaporation, atmospheric mixing, moisture recycling, sublimation, and/or relative humidity, but the data set is not well suited to quantitatively assess isotopic variability associated with each of these processes. The seasonal $\Delta^{\prime 17}O$ pattern, which is absent in *d*-excess and opposite in sign from $\delta^{18}O$, appears in other data sets globally; it showcases the influence of seasonality on $\Delta^{\prime 17}O$ values of precipitation and highlights the need for further systematic studies to understand variation in $\Delta^{\prime 17}O$ values of precipitation.

## 1. Introduction

Ratios of oxygen isotopes (^18^O/^16^O, or $\delta^{18}O$) are often used to reconstruct past environmental and climatic conditions (e.g., Koch, 1998; Liu et al., 2017; Rowley, 2007; Zachos et al., 2001). However, interpreting $\delta^{18}O$ data from geologic archives can be challenging as it is often difficult to attribute $\delta^{18}O$ variation to specific fractionating processes (e.g., Rech et al., 2019; Thompson et al., 2000; Wostbrock & Sharp, 2021). This challenge can be particularly problematic in terrestrial paleoclimate archives that often integrate information about temperature, seasonality, vegetation cover, evaporation, the amount and isotopic composition of local precipitation, atmospheric and oceanic conditions, and biological physiology (e.g., Breecker et al., 2009; Bryant & Froelich, 1995; Kelson et al., 2020; Kohn, 1996; Quade et al., 2007). Differentiating among these $\delta^{18}O$ controls is a critical component to improving reconstructions of past climate.

Recent advances in characterizing distributions of ^17^O, the third and least abundant stable isotope of oxygen, demonstrate potential for using ^17^O/^16^O ratios $\left(\delta^{17}O\right)$ to help constrain interpretations of $\delta^{18}O$ records (see recent reviews by Aron et al., 2021a; Galewsky et al., 2016; Passey & Levin, 2021; Surma et al., 2021). The power of $\delta^{17}O$ measurements comes from assessing how their distributions vary from expected relationships with $\delta^{18}O$ values. When used in studies of the hydrosphere (past and present), the $\Delta^{\prime 17}O$ parameter is defined as the deviation from a reference relationship between $\delta^{\prime 18}O$ and $\delta^{\prime 17}O$ (Barkan & Luz, 2007):

(1) $${\Delta^{\prime }}^{17}\text{O}={\delta^{\prime }}^{17}\text{O}-0.528*{\delta^{\prime }}^{18}\text{O}$$

where $\delta =\left(R_{\text{sample}}/R_{\text{standard}}-1\right)$, *R* is the ratio of heavy-to-light isotopes, and δ′ is the logarithmic version of $\delta \left(\delta^{\prime }=ln(\delta +1)\right.$; Miller, 2002). The slope of the $\delta^{\prime 18}O-\delta^{\prime 17}O$ reference relationship $\left(\lambda_{\text{ref}}\right)$, 0.528, was initially thought to approximate the global relationship through meteoric water $\delta^{\prime 18}O$ and $\delta^{\prime 17}O$ (Luz & Barkan, 2010; Meijer & Li, 1998), but recent studies indicate this value is biased high by polar waters (Aron et al., 2021a; Miller, 2018; Sharp et al., 2018). Still, we continue to use 0.528 in the definition of $\Delta^{\prime 17}O$ to maintain consistency with previous work; this value also has a mechanistic significance as it is nearly identical to the $\delta^{\prime 18}O-\delta^{\prime 17}O$ slope during Rayleigh distillation (Luz & Barkan, 2010).

The last several years have produced a spate of studies that showcase the utility of high-precision $\Delta^{\prime 17}O$ analysis for reconstructing past environments. This work has shown how $\Delta^{\prime 17}O$ data from sediments and fossils can be used to account for the effects of evaporation to reconstruct the $\delta^{18}O$ values of meteoric waters (e.g., Passey & Ji, 2019), identify shifts in paleohydrology (e.g., Evans et al., 2018; Gázquez et al., 2020), serve as a proxy for paleo-humidity (Alexandre et al., 2018; Gázquez et al., 2018; Lehmann et al., 2022; Sha et al., 2020), refine paleoaltimetry estimates (Chamberlain et al., 2021; Ibarra et al., 2021; Kelson et al., 2022), and detect diagenesis (Gehler et al., 2011; Levin et al., 2014; Sengupta & Pack, 2018; Wostbrock et al., 2020). In each of these examples, $\Delta^{\prime 17}O$ brings information beyond what can be determined with the analysis of $\delta^{18}O$ alone and helps expand the utility of oxygen isotopes for reconstructing climate, hydrology, and elevation in ancient systems.

The $\Delta^{\prime 17}O$ sensitivity to evaporation is well documented in geologic materials and waters (Aron et al., 2021a; Beverly et al., 2021; Evans et al., 2018; Gázquez et al., 2020, 2018; Herwartz et al., 2017; Ibarra et al., 2021; Li et al., 2017; Passey et al., 2014; Passey & Ji, 2019; Surma et al., 2015, 2018; Voigt et al., 2021). Understanding the $\Delta^{\prime 17}O$ variation in meteoric waters that are relatively unevaporated—these are the waters responsible for the majority of recharge to terrestrial water reservoirs (e.g., lakes, rivers, groundwater, soil water) and are assumed to reflect primarily equilibrium fractionation processes—is critical to these studies, but it is not well defined. Until recently, average meteoric water was thought to have a $\Delta^{\prime 17}O$ value of ~33 per meg (Luz & Barkan, 2010) and this value was used as a benchmark in some paleoclimate applications (e.g., Ibarra et al., 2021; Passey & Ji, 2019). However, recent compilations of water $\delta^{18}O$ and $\delta^{17}O$ data show that $\Delta^{\prime 17}O$ values of meteoric water are regionally variable and that many non-polar waters yield $\Delta^{\prime 17}O$ values less than 33 per meg (Aron et al., 2021a; He et al., 2021; Miller, 2018; Sharp et al., 2018). Still, uncertainty around an average $\Delta^{\prime 17}O$ value of meteoric water exists because so many surface water $\Delta^{\prime 17}O$ data sets focus on waters that experienced extensive evaporation (e.g., Aron et al., 2021a; Bershaw et al., 2020) and all existing precipitation $\Delta^{\prime 17}O$ data sets are from single sites (Affolter et al., 2015; Beverly et al., 2021; Gázquez et al., 2017; Gimenez et al., 2021; He et al., 2021; Landais et al., 2010; Surma et al., 2018; Tian et al., 2021; Tian & Wang, 2019; Uechi & Uemura, 2019).

Here, we present precipitation $\delta^{18}O$, *d*-excess, and $\Delta^{\prime 17}O$ data from 26 sites in the western and central United States and stream $\delta^{18}O$, *d*-excess, and $\Delta^{\prime 17}O$ data from the Willamette River Basin in western Oregon. The distribution of sample sites and collection times make this data set ill-suited for spatial or temporal analysis, but we use the data set to evaluate variation in precipitation $\Delta^{\prime 17}O$ values in the North America, compare stream and precipitation $\Delta^{\prime 17}O$ values, and begin to determine the range of amount-weighted precipitation $\Delta^{\prime 17}O$ values in North America.

## 2. Isotope Systematics

The utility of triple oxygen isotope ratios in paleoclimate and hydrologic applications relies on characterizing differences in the linear relationships between $\delta^{\prime 18}O$ and $\delta^{\prime 17}O$ during equilibrium and kinetic fractionation. This approach is similar to the framework used to infer climatic and hydrologic information from $\delta^{18}O$ and $\delta^{2}H$ values in which most meteoric waters plot on a line with a slope of 8, reflecting equilibrium fractionation (Craig, 1961; Dansgaard, 1964; Horita & Wesolowski, 1994; Majoube, 1971), and processes involving kinetic fractionation with a lower slope (~2.5–8; Brady & Hodell, 2021; Gonfiantini et al., 2018). Analogous to $\Delta^{\prime 17}O$, *d*-excess quantifies the deviation from a reference relationship (Dansgaard, 1964), where

(2) $$d-\text{excess}=\delta^{2}\text{H}-8*\delta^{18}\text{O}.$$

The *d*-excess parameter provides information on non-equilibrium processes and has been used extensively to characterize evaporation during evapotranspiration, moisture transport, and precipitation (see Bowen et al., 2019; Galewsky et al., 2016; Gat, 1996). The magnitude of *d*-excess is controlled mainly by relative humidity during kinetic fractionation (Craig & Gordon, 1965) and by temperature during equilibrium fractionation (Majoube, 1971).

Following similar principles as *d*-excess, $\Delta^{\prime 17}O$ values of water track hydrological processes because there are distinct $\delta^{\prime 18}O-\delta^{\prime 17}O$ relationships for equilibrium and kinetic fractionation. The $\delta^{\prime 18}O-\delta^{\prime 17}O$ slope is higher (0.529) during equilibrium fractionation (Barkan & Luz, 2005; Young et al., 2002) and lower during kinetic fractionation (0.5185–0.5188; Barkan & Luz, 2007; Hellmann & Harvey, 2020). Distinctions between the reference slope (0.528) and slopes associated with fractionation mean that $\Delta^{\prime 17}O$ is more sensitive to processes involving kinetic fractionation (e.g., diffusive effects during evaporation) than equilibrium fractionation and most rainout processes because these fractionation slopes are very close to the reference slope. Given a minimal sensitivity of $\Delta^{\prime 17}O$ to temperature, the combined use of $\Delta^{\prime 17}O$ and *d*-excess holds promise for characterizing variations in moisture source relative humidity and temperature (e.g., Uechi & Uemera, 2019) and for constraining rain re-evaporation (e.g., Landais et al., 2010). When evaporation drives isotopic fractionation, *d*-excess and $\Delta^{\prime 17}O$ co-vary linearly (e.g., Landais et al., 2010; Li et al., 2017; Surma et al., 2018). In other circumstances, the lack of a relationship between *d*-excess and $\Delta^{\prime 17}O$ has been used to identify processes such as mixing and recycling (e.g., Landais et al., 2010; Voigt et al., 2021). Studies of precipitation, vapor, and lakes show that the combination of *d*-excess and $\Delta^{\prime 17}O$ make a powerful tool for understanding hydrological and meteorological processes (e.g., Galewsky et al., 2016; Landais, Steen-Larsen, et al., 2012; Pierchala et al., 2022). It can be difficult to generate *d*-excess records in the geologic record, as $\delta^{18}O$ and $\delta^{2}H$ are rarely preserved in the same material (e.g., Evans et al., 2018), but $\Delta^{\prime 17}O$ records represent an opportunity to track evaporation in ancient waters (lakes, body waters, rivers, soils etc.). Constraints on the $\Delta^{\prime 17}O$ values of the starting water that feeds these water bodies are critical for this approach.

## 3. Materials and Methods

### 3.1. Sample Collection

**Figure 1.**
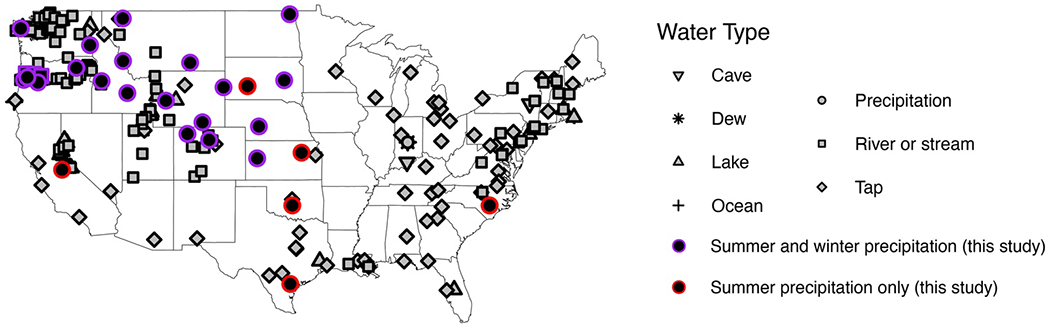
Spatial distribution of previously published (gray filled symbols) and new (black filled symbols) triple oxygen isotope water data from the United States.

**Figure 2.**
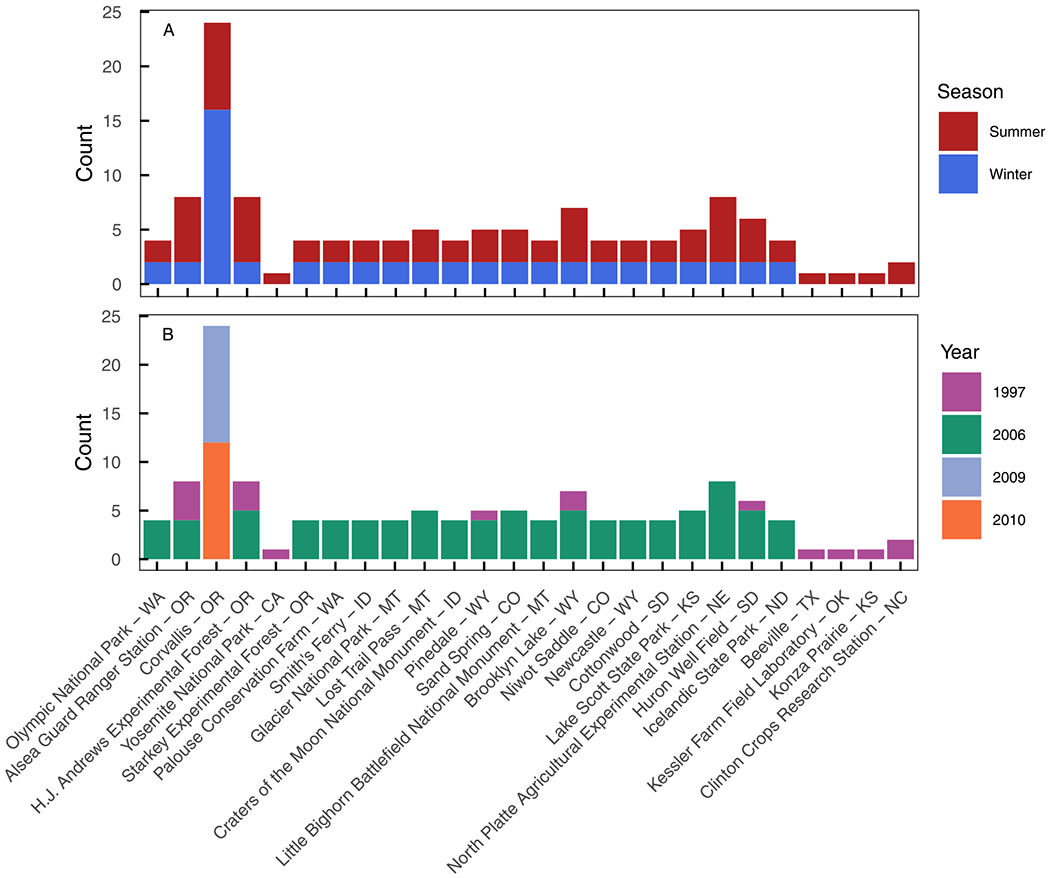
Histograms of seasonal (a) and annual (b) distributions of precipitation samples from this study. Sites are listed longitudinally with western-most sites (Washington and Oregon) on the left and the eastern-most site (North Carolina) on the right. The latitude and longitude of each site is reported in Table S1.

Precipitation and stream samples included in this study were selected for triple oxygen isotope analysis from two pre-existing sample sets. First, we selected 109 weekly precipitation samples from the USNIP data set (Welker, 2012) collected in 1997 (*n* = 19) and 2006 (*n* = 90) from 22 sites mostly in the western and central United States (Figures 1 and 2, Table S1). Samples were selected to explore the impacts of geography and season on $\Delta^{\prime 17}O$ variation in summer (June–August) and winter (December–February) months (Figure 2a). This data set provides an initial view of $\Delta^{\prime 17}O$ values of seasonal and annual amount-weighted precipitation in the western and central United States but is too spatially coarse to directly compare site-to-site or sample-to-sample data, characterize relationships between $\Delta^{\prime 17}O$ values with local conditions, or evaluate local $\Delta^{\prime 17}O$ variations during synoptic events.

Second, we analyzed $\delta^{18}O$ and $\delta^{17}O$ values of 24 weekly precipitation samples from Corvallis, Oregon, and 18 stream samples from the surrounding Willamette River Basin (Table S1; Brooks et al., 2012a, 2012b) to explore seasonal $\Delta^{\prime 17}O$ variability and compare $\Delta^{\prime 17}O$ values between streams and precipitation. The precipitation samples from Corvallis include one sample per month from February 2009 to December 2010. Stream samples were collected three times per year in 2009 and 2010 to capture spring snowmelt, low summer flow, and winter storms. Stream samples were collected from three small streams in an east-west transect across the Willamette River Basin that vary in distance from the Pacific Ocean and thus vary in precipitation rainout effects. The western most stream was a small stream within the Luckiamute River watershed, an eastward-facing basin that drains the Coast Range (*n* = 6, 121 m above sea level (masl)) and flows into the western side of the Willamette Valley. The other two small streams were located within North Santiam River watershed, a westward-facing basin that drains the Cascade Mountains (*n* = 12) and flows into the eastern side of the Willamette Valley. The two North Santiam stream sites varied in elevation (838 masl and 197 masl, *n* = 6 each).

### 3.2. Isotopic Analysis

#### 3.2.1. $\delta^{18}O$ and $\delta^{17}O$ Measurements

Triple oxygen isotope ratios of waters were analyzed using the cobalt(III) fluoride method developed by Baker et al. (2002) and Barkan and Luz (2005). Measurements were made on a Thermo Scientific MAT 253 isotope ratio mass spectrometer (IRMS) at Johns Hopkins University in 2012–2014, using methods described in Li et al. (2015, 2017) and Passey et al. (2014). All $\delta^{18}O$ and $\delta^{17}O$ values were normalized to the VSMOW-SLAP scale using the approach described by Schoenemann et al. (2013), using measurements of VSMOW2 and SLAP2 analyzed concurrently with unknowns. As such, values of $\delta^{18}O$ were defined as 0% for VSMOW2 and −55.5% for SLAP2, $\Delta^{\prime 17}O$ was assumed to be 0% for both VSMOW2 and SLAP2, $\lambda_{\text{ref}}$ was defined as 0.528, and $\delta^{17}O$ was 0% for VSMOW2 and −29.6986% for SLAP2. We monitored analytical performance by regularly analyzing $\delta^{18}O$ and $\delta^{17}O$ values of USGS 45, 46, 47, and 48 reference waters and determined that analytical precision (root-mean-square-error) of USGS waters was better than 0.2% for $\delta^{17}O$, 0.3% for $\delta^{18}O$, and 7 per meg for $\Delta^{\prime 17}O$. See Table S2 for reports of raw and normalized data for standards and unknowns.

#### 3.2.2. $\delta^{18}O$ and $\delta^{2}H$Data

The $\delta^{18}O$ and $\delta^{2}H$ data reported in this study are considered part of the primary data set, but were previously published in Brooks et al. (2012a, 2012b) and Welker (2012). USNIP precipitation samples were analyzed at the University of Alaska Anchorage Stable Isotope Lab with a TCEA unit attached to a Thermo Finnigan IRMS (Welker, 2012). Analytical precision of the measurements at the University of Alaska Anchorage (UAA) are 0.2% for $\delta^{18}O$ and 0.5% for $\delta^{2}H$. Stream samples from the Willamette River Basin and precipitation samples from Corvallis, OR were analyzed on a Laser Absorption Water-Vapor Isotope Spectrometer (Los Gatos Research (LGR) Model 908-0004) at the Integrated Stable Isotope Research Facility at the Western Ecology Division of the Environmental Protection Agency (EPA), Corvallis, OR (Brooks et al., 2012a, 2012b). Analytical precision of $\delta^{18}O$ and $\delta^{2}H$ values from the EPA measurements are 0.2 and 0.5%, respectively (Brooks et al., 2012a, 2012b). All $\delta^{18}O$ and $\delta^{2}H$ data from UAA and the EPA research facility are reported relative to VSMOW.

#### 3.2.3. Data Quality Checks and Caveats

Precipitation and stream samples were stored for up to 15 yr before triple oxygen isotope analysis, so it is important to evaluate sample quality. At EPA, precipitation and stream samples were stored upside-down in 20 ml glass scintillation vials with polycone caps. USNIP samples were stored at UAA in 40 ml screw cap Nalgene bottles at 4°C. These common storage techniques typically preserve the isotopic composition of water samples, but it is important to confirm that isotopic ratios did not drift during storage.

First, to confirm that isotopic ratios did not drift and to evaluate analytical accuracy, we compared the $\delta^{18}O$ values measured at Johns Hopkins University with those measured at the EPA research facility or UAA. More than 98% of the $\delta^{18}O$ values measured at Johns Hopkins University are identical (within $\delta^{18}O$ analytical precision) of those analyzed at EPA or UAA (Figure S1 in Supporting Information S1). Two precipitation samples have $\delta^{18}O$ values that differ by more than 4%. We can find no clear analytical explanation for such different $\delta^{18}O$ values. Isotope data from these outliers are reported in Table S1 but are excluded from subsequent analysis.

Second, because the goal of this study is to explore the variability of $\Delta^{\prime 17}O$ across the western and central United States, we ensured that our data set is representative of isotopic compositions in this region. To confirm this, we compared the $\delta^{18}O$ and *d*-excess values from our data set with previously published data from the western and central United States (Figure S2 in Supporting Information S1) accessed from the University of Utah water isotope database (waterisotopesdb.org; Putman & Bowen, 2019). Both the range and patterns of $\delta^{18}O$ and *d*-excess values from our data set are statistically indistinguishable from previously published observations (Welch two sample *t* test *p* values > 0.05), so we conclude that our data set is representative of isotopic variability across the western and central United States.

Third, we confirmed the accuracy and precision of our $\Delta^{\prime 17}O$ measurements by comparing $\Delta^{\prime 17}O$ values of USGS reference waters measured at Johns Hopkins University with other reported values of the same waters (Table S3 in Supporting Information S1). Values of $\Delta^{\prime 17}O$ of USGS reference waters reported in this study are statistically indistinguishable from those reported by Aron et al. (2021a) and Berman et al. (2013), so we are confident that the $\Delta^{\prime 17}O$ data reported in this study are accurate and precise.

In total, we analyzed $\delta^{18}O$ and $\delta^{17}O$ from 151 water samples. Excluding the two precipitation samples with very different (more than 4%) $\delta^{18}O$ values between Johns Hopkins University and EPA or UAA, the final data set contains 149 samples (18 stream and 131 precipitation samples).

### 3.3. Meteorological Data

Weekly precipitation amount data were collected at each USNIP site as part of the North American Deposition Program (NADP; http://nadp.slh.wisc.edu/) and at the EPA Western Ecology Division climate station in Corvallis, OR (Brooks et al., 2012a, 2012b). The EPA climate station also recorded temperature and relative humidity. Temperature and relative humidity were not recorded as part of the NADP network, so these data were filled in from nearby National Weather Service meteorological stations from the MesoWest database (https://mesowest.utah.edu/).

### 3.4. Theoretical Modeling

Simple Rayleigh distillation and evaporation modeling was conducted to compare theoretical $\Delta^{\prime 17}O,\delta^{18}O$, and *d*-excess values during Rayleigh distillation and pan evaporation with observed precipitation $\Delta^{\prime 17}O,\delta^{18}O$, and *d*-excess data. Theoretical values were calculated using supplementary script 4 from Aron et al. (2021a) and average meteorological conditions from coastal and near-coastal sites in this study (Olympic National Park, Alsea Guard Ranger Station, H.J. Andrews Experimental Forest, and Corvallis, OR) as the initial conditions for calculations.

## 4. Results

### 4.1. General Results

**Figure 3.**
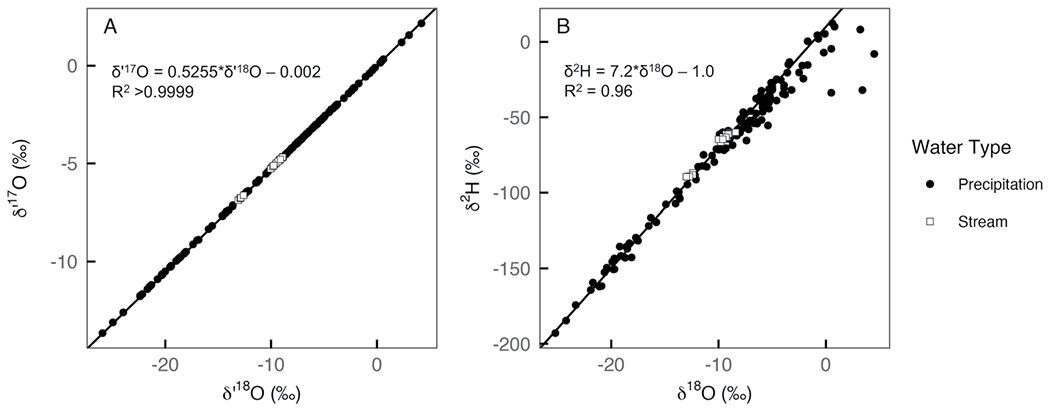
Scatterplots of precipitation (filled circles) and stream (open squares) $\delta^{\prime 18}O$ versus $\delta^{\prime 17}O$ (a) and $\delta^{18}O$ versus $\delta^{2}H$ (b) from this study. The solid black lines show meteoric water reference lines with slopes of 0.528 and 8 and intercepts of 0 and 10, respectively.

Site information, meteorological data, and isotope data are reported in Table S1. Raw isotope data are reported in Table S2. Values of $\delta^{18}O$ ranged from −25.2 to 45%, $\delta^{17}O$ ranged from −13.6 to 2.2%, $\delta^{2}H$ ranged from −192.9 to 12.3%, *d*-excess ranged from −59.1 to 17.8%, and $\Delta^{\prime 17}O$ ranged from −54 to 71 per meg. As expected, $\delta^{\prime 17}O$ and $\delta^{\prime 18}O$ were strongly correlated (*R*_2_ > 0.9999), following a line with a slope 0.5255 ± 0.0002 and intercept −0.002 ± 0.002 (uncertainty on the reported slopes and intercepts is the standard error) that is slightly shallower than the triple oxygen isotope reference line $\left(\delta^{\prime 17}O=0.528^{*}\delta^{\prime 18}O\right.$; Luz & Barkan, 2010; Figure 3a). Values of $\delta^{18}O$ and $\delta^{2}H$ were also well correlated (*R*^2^ = 0.96) with most points on or slightly below the $\delta^{18}O-\delta^{2}H$ Global Meteoric Water Line (Craig, 1961). The regression line through observed $\delta^{18}O$ and $\delta^{2}H$ values had a slope of 7.2 ± 0.1 and intercept of −1.0 ± 1.3 (Figure 3b).

### 4.2. Precipitation

**Figure 4.**
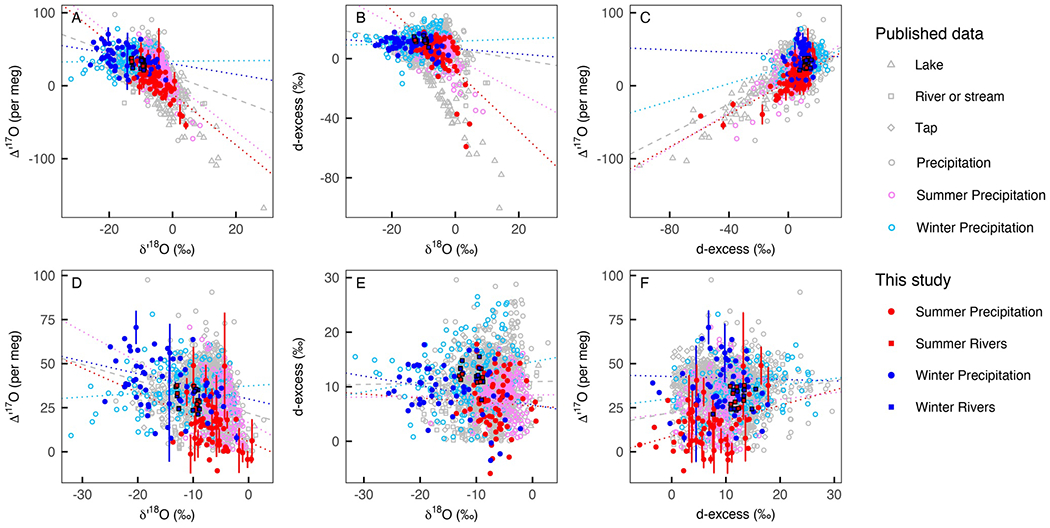
Scatterplots of $\delta^{\prime 18}O$ versus $\Delta^{\prime 17}O$ (a and d), $\delta^{18}O$ versus *d*-excess (b and e), and *d*-excess versus $\Delta^{\prime 17}O$ (c and f). Error bars on $\Delta^{\prime 17}O$ data show the standard deviation of $\Delta^{17}O$ measurements. Shape differentiates water types and color differentiates seasons. In our study, summer data are in red and winter data are in blue. Among the published data, summer precipitation data are in pink and winter precipitation data are in teal. Precipitation data from spring or fall, or from tropical regions with no clear seasonal climate patterns, are in gray. New surface water data (red from summer, blue from winter) reported in this study are outlined in black. The dotted lines show regression lines through summer (red and pink) and winter (blue and teal) datasets. Panels (a–c) show all the available data points (published studies and this study); panels (d–f) only show data points with positive *d*-excess and positive $\Delta^{\prime 17}O$ values. In each panel, new data reported in this study are shown with solid symbols and previously published data are shown with open symbols. Published precipitation data are from Affolter et al. (2015), Beverly et al. (2021), Gázquez et al. (2017), Gimenez et al. (2021), He et al. (2021), Landais et al. (2010), Luz and Barkan (2010), Surma et al. (2018), Tian et al. (2021, 2019), and Uechi and Uemura (2019). Published river or stream data are from Affolter et al. (2015), Aron et al. (2021a), Bergel et al. (2020), Bershaw et al. (2020), Beverly et al. (2021), Luz and Barkan (2010), Nava-Fernandez et al. (2020), Passey and Ji (2019), Surma et al. (2015), and Voigt et al. (2021). Published tap water are from Aron et al. (2021a), Li et al. (2015, 2017), Luz and Barkan (2010), and Tian et al. (2019). Published lake data are from Aron et al. (2021a), Bershaw et al. (2020), Beverly et al. (2021), Li et al. (2017), Luz and Barkan (2010), Passey and Ji (2019), Surma et al. (2015), Surma et al. (2018), and Voigt et al. (2021).

**Figure 5.**
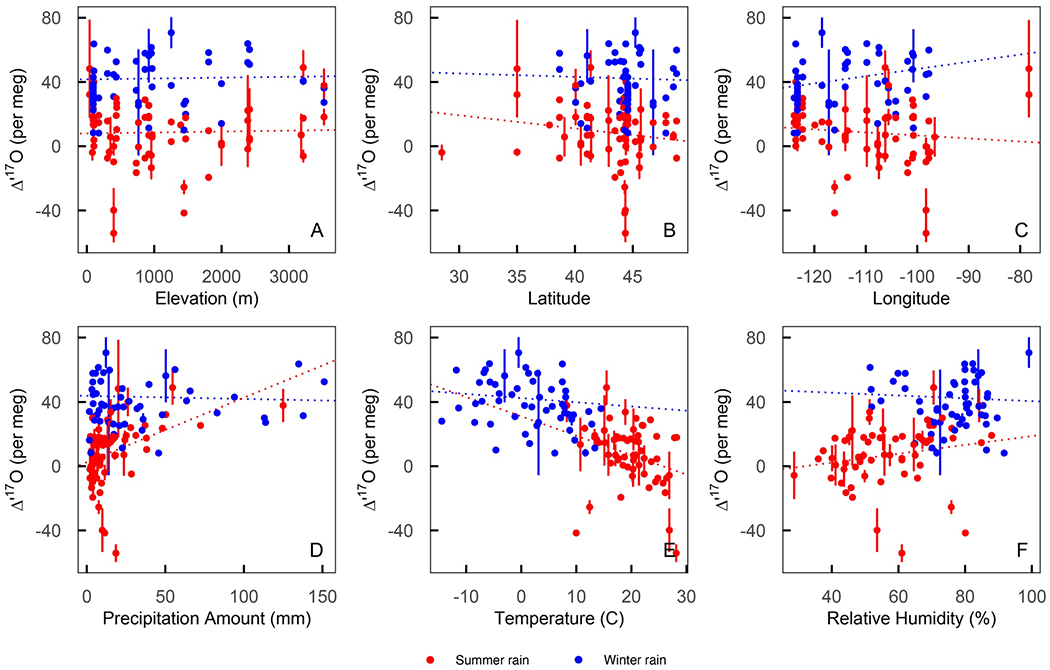
Scatterplots of summer (red) and winter (blue) precipitation $\Delta^{\prime 17}O$ versus elevation (a), latitude (b), longitude (c), precipitation amount (d), local average weekly temperature (e), and local average weekly relative humidity (f). The dotted lines show linear regression lines between $\Delta^{\prime 17}O$ and each *x*-axis variable for summer (red) and winter (blue) data.

Annual amount-weighted average precipitation $\Delta^{\prime 17}O$ was 31 per meg. Among the precipitation samples, the best-fit linear regression lines were $\delta^{\prime 17}O=0.5255\pm 0.0002*\delta^{\prime 18}O-0.002\pm 0.0002$ and $\delta^{2}H=7.2\pm 0.1*\delta^{18}O-1.4\pm 1.4$. Precipitation $\Delta^{\prime 17}O$ values were strongly negatively correlated with $\delta^{\prime 18}O$ (Pearson’s *r* = −0.72, *p* < 0.05, Figure 4a) and strongly positively correlated with *d*-excess (*r* = 0.63, *p* < 0.05, Figure 4c). The negative correlation between precipitation *d*-excess and $\delta^{18}O$ (*r* = −0.48, *p* < 0.05, Figure 4b) was weaker than that between $\Delta^{\prime 17}O$ and $\delta^{18}O$; much of this negative correlation for *d*-excess and $\delta^{18}O$ was related to a handful of samples with low (<0%) *d*-excess values (Figure S3 in Supporting Information S1). Excluding low *d*-excess samples, precipitation *d*-excess and $\delta^{18}O$ were only weakly correlated (*r* = −0.29, *p* < 0.05, Figure 4e) while $\Delta^{\prime 17}O$ and $\delta^{\prime 18}O$ remained strongly negatively correlated (*r* = −0.64, *p* < 0.05, Figure 4d). Similarly, correlations between $\delta^{18}O,\Delta^{\prime 17}O$, and *d*-excess and local meteorological conditions such as precipitation amount (*r* = − 0.20, 0.34, and 0.28, respectively), temperature 0.83, −0.69, −0.31, respectively), and relative humidity (*r* = −0.45, 0.49, 0.17, respectively) were generally stronger for $\delta^{18}O$ and $\Delta^{\prime 17}O$ than for *d*-excess (Figure 5, Figure S4 in Supporting Information S1). We used meteorological quarters to consider seasonal patterns, where winter was defined as the months of December-January-February and summer is June-July-August (Table S1). Most precipitation samples in the data set were collected during winter or summer so we focused our comparison on these seasons.

**Figure 6.**
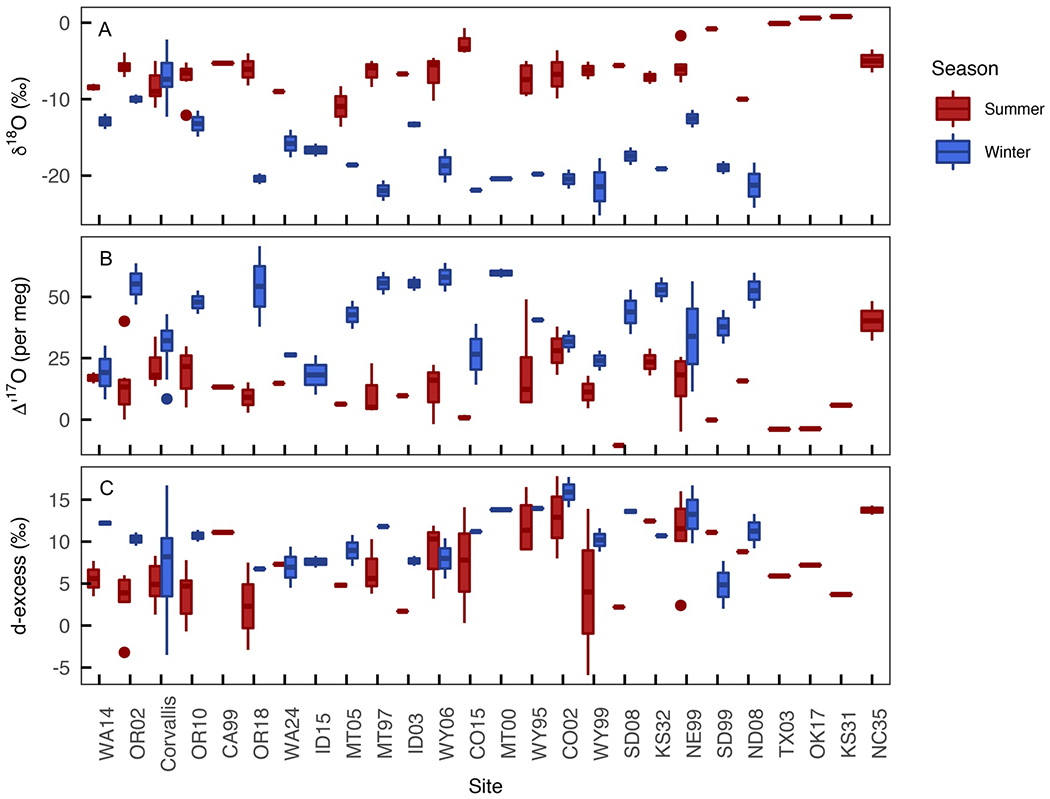
Box plots of summer (red) and winter (blue) precipitation $\delta^{18}O(a),\Delta^{\prime 17}O(b)$, and *d*-excess (c). Sites are listed longitudinally with western-most sites (Washington and Oregon) on the left and the eastern-most site (North Carolina) on the right. The evaporated precipitation samples are excluded from this figure to highlight seasonal variation and reduce the isotopic ranges. A version of this figure that includes all the precipitation data is included in the Supporting Information (Figure S6 in Supporting Information S1).

The most pronounced pattern among the precipitation data was seasonal $\delta^{18}O$ and $\Delta^{\prime 17}O$ variability (Figures 4–6). Across all the years of sample collection and most sites, seasonal amount-weighted precipitation $\Delta^{\prime 17}O$ averages were higher in the winter (40 ± 15 per meg), lower in the summer (18 ± 18 per meg), and statistically distinct (*p* < 0.05, Figure 4). The seasonal pattern of precipitation $\delta^{18}O$ was opposite, with lower amount-weighted $\delta^{18}O$ in the winter (−13.0 ± 5.9%) than the summer (−7.0 ± 2.9%). Average seasonal amount-weighted summer and winter *d*-excess values were nearly indistinguishable (7.0 ± 12.4% and 10.7 ± 4.6%, respectively). These seasonal $\delta^{18}O$ and $\Delta^{\prime 17}O$ patterns were consistent across almost every site but were slightly less pronounced along the Pacific coast and in the Willamette River Basin where the isotopic composition of rain was presumably more closely tied to oceanic moisture source conditions than sites located in the continental interior (Figure 6). Regression lines for $\delta^{\prime 18}O-\delta^{\prime 17}O$ and $\delta^{18}O-\delta^{2}H$ also varied seasonally, with steeper slopes and higher intercepts in the winter than in the summer (Figure S5 in Supporting Information S1).

**Figure 7.**
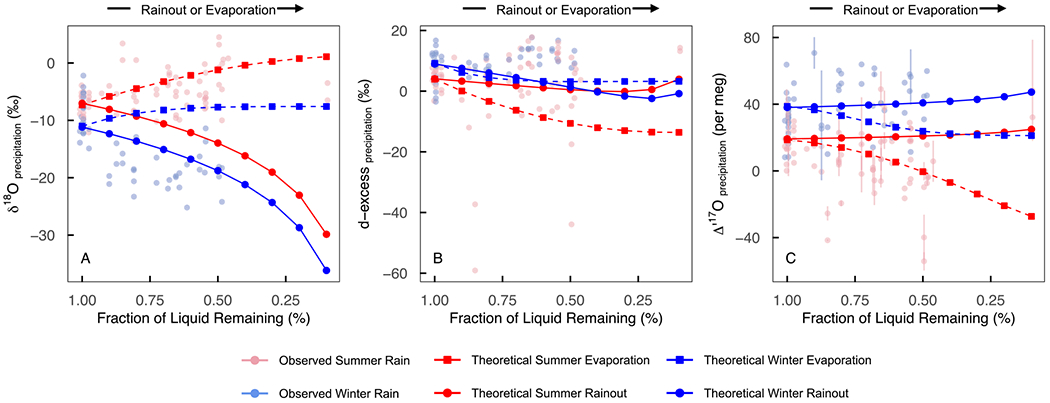
Scatterplots of theoretical $\delta^{18}O(a),d$-excess (b), and $\Delta^{\prime 17}O$ (c) undergoing rainout Rayleigh Distillation (filled circles) and pan evaporation (filled squares) in winter (blue) and summer (red) seasons. Initial temperature and relative humidity were 7°C and 83% in the winter and 17°C and 65% in the summer. The pale symbols show observed summer (red) and winter (blue) precipitation data reported in this study. The observed data are plotted versus longitude, with the western-most sites on the left corresponding to coastal air masses that have not lost much moisture and the eastern-most sites on the right corresponding to air masses that have lost much of their moisture. Observations are included in these plots to help contextualize the outputs from these simple modeling exercises in general terms; they are not intended for direct comparison.

This data set does not show clear spatial patterns in precipitation $\Delta^{\prime 17}O$ values. Correlations were weak between precipitation $\delta^{18}O,\Delta^{\prime 17}O$, and *d*-excess with elevation (*r* = −0.21, 0.02, 0.13, respectively), latitude (*r* = −0.24, 0.05, −0.16, respectively), and longitude (*r* = 0.01, −0.11, 0.07, respectively; Figure 5). However, a seasonal difference was found across the Cascade Range, with less seasonal $\delta^{18}O$ and $\Delta^{\prime 17}O$ variability at sites west of the Cascades (OR02, Corvallis, OR10) and pronounced seasonal distinctions at sites east of the Cascades (OR18, Figure 6, Figure S6 in Supporting Information S1). This longitudinal pattern was absent or even slightly reversed for *d*-excess, which had slightly larger differences (generally > 6.5%) between summer and winter values at sites west of the Cascades and smaller seasonal differences (generally < 5%) at sites east of the Cascades (Figure 6c). Overall, $\Delta^{\prime 17}O$ was generally more variable at inland sites than those closer to the Pacific Coast, but proximity to the coast is not a reliable predictor of $\Delta^{\prime 17}O$ variability (Figures 5 and 6). Theoretical modeling further confirms that isotopic signals of west-to-east rainout across the western and central United States are not clearly captured in observed precipitation $\Delta^{\prime 17}O,\delta^{18}O$, and *d*-excess data (Figure 7). Still, the small size of this data set limited exploration of trends in isotopic variation between sampling years or events, within sites, or at a greater spatial resolution.

### 4.3. Streams

The isotopic compositions of streams (*n* = 18) in the Willamette River Basin ranged from −13.0 to −8.4% for $\delta^{18}O$, −6.9 to −4.7% for $\delta^{17}O$, −89.5 to −59.9% for $\delta^{2}H$, 7.6–15.5% or *d*-excess, and 21 to 37 per meg for $\Delta^{\prime 17}O$. Average $\Delta^{\prime 17}O$ and $\delta^{18}O$ values for the Luckiamute and North Santiam Rivers (30 ± 6 per meg and −10.4 ± 1.7%, respectively) were statistically indistinguishable from the annual amount-weighted average value of precipitation in Corvallis (29 ± 9 per meg and −7.8 ± 2.6%). The best-fit linear regressions through the stream samples were $\delta^{\prime 17}O=0.5272\pm 0.0008*\delta^{\prime 18}O+0.022\pm 0.008$ and $\delta^{2}H=7.52\pm 0.2*\delta^{18}O+6.4\pm 2.5$ (Figure 3).

Average *d*-excess and $\Delta^{\prime 17}O$ values were slightly higher (12.6% and 32 per meg, respectively) in the North Santiam streams than for the stream within the Luckiamute River Basin (11.1% and 27 per meg, respectively). However, site-specific isotopic compositions were statistically indistinguishable from each other (*p* values > 0.05) and there were no clear trends between stream $\Delta^{\prime 17}O$ or *d*-excess along a longitudinal transect (*r* = 0.31 and 0.44, respectively). Stream $\delta^{18}O,\Delta^{\prime 17}O$, and *d*-excess values exhibited no seasonal pattern (Figure 4). Stream $\delta^{18}O$ variation was strongly negatively correlated with elevation (*r* = −0.990), with lower $\delta^{18}O$ values (−13.0 to −12.4%) from the high elevation stream on the North Santiam River and higher $\delta^{18}O$ values (−10.0 to −8.4%) from the stream in the Luckiamute watershed and the low elevation stream in the North Santiam basin.

## 5. Discussion

### 5.1. $\Delta^{\prime 17}O$ Observations in Context of Prior Studies

In many ways, the observations presented here confirm trends observed of $\Delta^{\prime 17}O,\delta^{\prime 18}O,\delta^{2}H$, and *d*-excess data from precipitation and streams in other mid- and low-latitude regions (e.g., Bershaw et al., 2020; Marchetti & Marchetti, 2019). This growing body of work shows similarities among seasonal distinctions, isotopic relationships between precipitation and stream water, and the precipitation $\delta^{\prime 18}O-\delta^{\prime 17}O$ regression slope (Figure 4). We also do not observe relationships between either precipitation or stream $\Delta^{\prime 17}O$ values and elevation, precipitation amount, latitude, longitude, or local meteorological conditions (Figure 5), which is consistent with previous work (e.g., Aron et al., 2021a). It is possible that spatial $\Delta^{\prime 17}O$ relationships exist, they just are not evident in this data set largely due to the distribution of samples (i.e., samples were collected from different sites at different times and recorded different precipitation events). Additional work to investigate spatial $\Delta^{\prime 17}O$ trends is still needed. Despite this limitation, the new data presented here and the existing body of work makes clear two important points: seasonal distinctions in $\Delta^{\prime 17}O$ values of precipitation are evident and they are consistent across a variety of geographic and climate regions (see discussion in Section 5.3).

Our observation that the $\Delta^{\prime 17}O$ values of stream water in the northwestern U.S. are seasonally invariant and generally less variable than those of precipitation is also consistent with previous studies (Figures 3 and 4). The relatively narrow range of stream $\Delta^{\prime 17}O$ values occurs because streams, especially in regions where water supplies are dominated by snowmelt and groundwater-fed recharge, typically integrate annual conditions and reflect the annual amount-weighted average isotopic composition of precipitation (Dutton et al., 2005; Kendall & Coplen, 2001). Streams can have very low $\Delta^{\prime 17}O$ values (<~−20 per meg ) and a larger $\Delta^{\prime 17}O$ range than that of precipitation, but these values typically occur in arid regions where slow-flowing streams experience a high degree of evaporation (e.g., Surma et al., 2015; Voigt et al., 2021).

The precipitation $\delta^{\prime 18}O-\delta^{\prime 17}O$ regression slope (0.5255) in this data set is lower than the reference value (0.5255); Luz & Barkan, 2010) but is also consistent with previous precipitation observations (Table S4 in Supporting Information S1). This highlights two important points. First, nearly every precipitation $\delta^{\prime 18}O-\delta^{\prime 17}O$ slope is less than 0.528. Second, on seasonal timescales, nearly every previous study of precipitation and water vapor has reported higher $\delta^{\prime 18}O-\delta^{\prime 17}O$ regression slopes in the winter and lower $\delta^{\prime 18}O-\delta^{\prime 17}O$ regression slopes in the summer (Affolter et al., 2015; Gimenez et al., 2021; He et al., 2021; Surma et al., 2021; Tian et al., 2018; Tian & Wang, 2019; Uechi & Uemura, 2019). This seasonal pattern leads to higher $\Delta^{\prime 17}O$ values in the winter and lower $\Delta^{\prime 17}O$ values in the summer (Figure 4a). This suggests that (*a*) the $\delta^{\prime 18}O-\delta^{\prime 17}O$ relationship of most precipitation samples differs from the reference relationship and (b) that precipitation $\delta^{\prime 18}O$ and $\delta^{\prime 17}O$ values record more than just Rayleigh distillation. Considering that 0.529 is the theoretical $\delta^{\prime 18}O-\delta^{\prime 17}O$ slope for equilibrium fractionation, 0.518 is the theoretical slope for kinetic fractionation, and Rayleigh distillation produces slopes of 0.528, the consistent observation that precipitation $\delta^{\prime 18}O-\delta^{\prime 17}O$ slopes are less than 0.528 suggests that $\delta^{\prime 18}O-\delta^{\prime 17}O$ relationships hold information about both Rayleigh and non-Rayleigh related processes (Aron et al., 2021a; Luz & Barkan, 2010).

### 5.2. Controls on Precipitation $\Delta^{\prime 17}O$ in the Western and Central United States

The consistency among this data set and other triple oxygen isotope studies of precipitation (i.e., precipitation $\delta^{\prime 18}O-\delta^{\prime 17}O$ slopes <0.528, seasonal distinctions in precipitation $\Delta^{\prime 17}O$) suggests systematic controls on $\Delta^{\prime 17}O$, but the fractionating processes responsible for this variation have yet to be conclusively identified. In the next sections, we explore the processes and conditions that may be responsible for the relationships we observe among $\delta^{18}O$, *d*-excess, and $\Delta^{\prime 17}O$ values in the western and central United States.

#### 5.2.1. Evaporation

Surface and sub-cloud evaporation are the most well studied processes in the triple oxygen isotope literature. This is likely because the magnitude of $\Delta^{\prime 17}O$ variability due to evaporation is often much greater than the analytical precision of $\Delta^{\prime 17}O$ measurements, evaporation can be hard to identify with $\delta^{18}O$ alone, isotopic models of evaporation are well established, and the co-variation of $\delta^{18}O,\Delta^{\prime 17}O$, and *d*-excess during evaporation is relatively easy to identify. This co-variation includes a negative correlation between $\delta^{18}O$ and $\Delta^{\prime 17}O$ or *d*-excess, a positive correlation between *d*-excess and $\Delta^{\prime 17}O$, and *d*-excess-$\Delta^{\prime 17}O$ slopes from ~0.7 to 2 per meg %^−1^ (e.g., Barkan & Luz, 2007; Landais et al., 2010; Li et al., 2017; Luz & Barkan, 2010).

We observe two signals of sub-cloud evaporation in our precipitation data set. First, a clear signal of evaporation was found among a small (*n* = 13) subset of summer precipitation samples that have positive $\delta^{18}O$ values, negative $\Delta^{\prime 17}O$ values, negative *d*-excess values, a strong positive correlation between $\Delta^{\prime 17}O$ and *d*-excess (*r* = 0.82), and a *d*-excess $-\Delta^{\prime 17}O$ slope of 0.7 ± 0.2 per meg %^−1^ (Figure 4a–4c and Figure S3 in Supporting Information S1). Such low (>~ −10%) *d*-excess values are unusual for precipitation, but are occasionally observed in western and central United States precipitation (Figure S2 in Supporting Information S1) due to sub-cloud evaporation (e.g., Marchetti & Marchetti, 2019). Evaporation might also have occurred after samples accumulated in the rain collection bucket as these summer precipitation events were small in amount, but this is unlikely because NADP has verified that collection devices essentially eliminate evaporative water loss (Lynch et al., 1996). Second, a weaker signal of evaporation occurred among all the summer rain samples. This evaporation was inferred from seasonal $\delta^{\prime 18}O-\delta^{\prime 17}O$ and $\delta^{18}O-\delta^{2}H$ regression lines (Figure S5 in Supporting Information S1). A strong positive correlation (*r* = 0.75) and positive slope (1.1 per meg %^−1^) between summer $\Delta^{\prime 17}O$ and *d*-excess and a slight positive correlation between summer $\Delta^{\prime 17}O$ values and local relative humidity (*r* = 0.19) support this interpretation (Gimenez et al., 2021; Landais et al., 2010). These signals are most likely related to sub-cloud evaporation during small summer storms (e.g., Benjamin et al., 2004; Eastoe & Dettman, 2016; Friedman et al., 2002; Marchetti & Marchetti, 2019). Theoretical evaporation modeling (Figure 7) confirms this and shows that evaporation may explain the isotope ratios of a handful of precipitation samples in this data set, but was not the single controlling mechanism that drives the variation in $\Delta^{\prime 17}O$ values in this study.

#### 5.2.2. Relative Humidity

Previous work has shown that precipitation $\Delta^{\prime 17}O$ can reflect variations of relative humidity above oceanic moisture sources, along moisture trajectories, and/or at local sample collection sites (e.g., Landais, Steen-Larsen, et al., 2012; Surma et al., 2021; Uechi & Uemura, 2019), but a clear relative humidity-$\Delta^{\prime 17}O$ relationship is not observed in this data set. Similarly, precipitation *d*-excess value and local relative humidity are weakly correlated (*r* = 0.17) in this data set. The absence of a relationship between $\Delta^{\prime 17}O$ and relative humidity may be related to terrestrial water cycling and the interior continental position of many of the sample sites (Fiorella et al., 2018) and/or composite weekly precipitation samples that are not clearly linked to site-specific average weekly relative humidity values.

#### 5.2.3. Rainout

Much like *d*-excess, $\Delta^{\prime 17}O$ is generally insensitive to rainout and Rayleigh distillation because these fractionating processes result in $\delta^{\prime 18}O-\delta^{\prime 17}O$ slopes that are nearly identical to the slope of the reference line (0.528, Equation 1). As a result, $\delta^{\prime 18}O$ and $\delta^{\prime 17}O$ variation during rainout occurs along a line that is offset from but essentially parallel to the reference line and $\Delta^{\prime 17}O$ values remain nearly constant (Aron et al., 2021a; Luz & Barkan, 2010). In our data set, regardless of season, weak annual correlations between $\Delta^{\prime 17}O$ and local precipitation amount (*r* = 0.34, Figure 5d), elevation (*r* = 0.02, Figure 5a), latitude (*r* = 0.05, Figure 5b), and longitude (*r* = −0.11, Figure 5c) indicate that rainout played a small role in the observed $\Delta^{\prime 17}O$ variability. Simple modeling of $\Delta^{\prime 17}O,\;\delta^{18}O$, and *d*-excess during Rayleigh distillation (Figure 7) further demonstrates this point.

#### 5.2.4. Sublimation, Stratospheric Intrusions, and Supersaturation

These controls are combined because although they all influence precipitation $\Delta^{\prime 17}O$ values (e.g., Schoenemann et al., 2014; Surma et al., 2021; Winkler et al., 2012), they likely play a small role in the observed variation. First, sublimation increases $\Delta^{\prime 17}O$ and *d*-excess values if precipitation condenses from sublimated vapor (Surma et al., 2021), but the lack of seasonal trends in *d*-excess values (Figure 4) means that sublimation is unlikely to be responsible for the high winter $\Delta^{\prime 17}O$ values that we observe. Second, stratospheric intrusions could increase precipitation $\Delta^{\prime 17}O$ values without affecting *d*-excess values by bringing water vapor with exceptionally high (> 1,000 per meg) $\Delta^{\prime 17}O$ values into the troposphere (Franz & Röckmann, 2005; Y. Lin et al., 2013; Winkler et al., 2012). However, this is unlikely because (a) stratospheric air is extremely dry and likely contributes very little to near-surface water cycles, (b) the high winter tropopause above North America generally limits stratospheric downdrafts, and (c) near-surface ozone levels, which increase during stratospheric intrusions, were low during the time periods when precipitation samples were collected (Cooper et al., 2012; M. Lin et al., 2015; Miller, 2013). Lastly, precipitation $\Delta^{\prime 17}O$ variability has been linked to supersaturation (e.g., Landais, Steen-Larsen, et al., 2012; Schoenemann et al., 2014). We consider this an unlikely explanation for our observations because the magnitude of supersaturation needed for observable fractionation is most common in polar regions where temperatures are very low (>~ −20°C). Further, supersaturation decreases $\Delta^{\prime 17}O$ values, which is opposite of the wintertime trends that we observe.

### 5.3. Seasonal Variability of Precipitation $\Delta^{\prime 17}O$

Seasonal distinctions in precipitation $\Delta^{\prime 17}O$ values are the most pronounced pattern in our data set; we observe higher $\Delta^{\prime 7}O$ values in the winter and lower $\Delta^{\prime 7}O$ values in the summer (Figures 4–6). Similar seasonal distinctions have also been observed in tropical precipitation in north central Africa (Landais et al., 2010) and eastern Singapore (He et al., 2021), mid-latitude precipitation in northwestern Switzerland (Affolter et al., 2015), southern Japan (Uechi & Uemura, 2019), central United States (Tian et al., 2018), and northern Spain (Gimenez et al., 2021), and polar precipitation in Greenland (Landais, Steen-Larsen, et al., 2012) and East Antarctica (Landais, Ekaykin, et al., 2012; Pang et al., 2019; Schoenemann & Steig, 2016; Touzeau et al., 2016). Seasonal $\Delta^{\prime 17}O$ variation has also been observed in tap water from the United States (Li et al., 2015) and atmospheric water vapor from central Europe (Surma et al., 2021).

Although seasonal $\Delta^{\prime 17}O$ variation has been observed across a wide range of climates and water types around the globe, explanations of this pattern vary widely. Seasonal precipitation $\Delta^{\prime 17}O$ variation is often explained by a switch from processes with a greater influence of kinetic fractionation in the summer and to those dominated by equilibrium fractionation in the winter (e.g., Affolter et al., 2015; Landais, Ekaykin, et al., 2012; Tian et al., 2018), but these explanations are not linked to climate conditions or hydrologic processes. In some instances, the seasonal $\Delta^{\prime 17}O$ pattern is directly related to the relative humidity at remote moisture sources (e.g., Landais, Steen-Larsen, et al., 2012; Uechi & Uemura, 2019), while in other cases seasonal $\Delta^{\prime 7}O$ variation is independent of relative humidity at either moisture source regions or sample collection sites (e.g., He et al., 2021; Li et al., 2015). In the tropics and mid-latitudes, seasonal $\Delta^{\prime 17}O$ variation has been linked to upstream moisture recycling (Tian et al., 2018), local raindrop re-evaporation (Gimenez et al., 2021; Landais et al., 2010), and convection tied to ENSO and regional monsoons (He et al., 2021). In snow-covered regions, sublimation can increase the $\Delta^{\prime 17}O$ of water vapor that is transported away from a snowpack, increasing the $\Delta^{\prime 17}O$ value of downstream precipitation (Pang et al., 2019; Surma et al., 2021). In polar regions, seasonal $\Delta^{\prime 17}O$ variations have been linked to the local precipitation rate at collection sites; relative humidity, sea surface temperatures, and the extent of sea ice at oceanic moisture sources; and kinetic fractionation during condensation under very cold, supersaturated conditions (Landais et al., 2008; Landais, Ekaykin, et al., 2012; Landais et al., 2012; Pang et al., 2019; Schoenemann & Steig, 2016; Schoenemann et al., 2014; Winkler et al., 2012).

The seasonal variation in $\Delta^{\prime 17}O$ that we observed in our data set likely reflects a combination of the processes listed above. Some of the low $\Delta^{\prime 17}O$ values from summer-time precipitation may result from post-condensation evaporation (e.g., Eastoe & Dettman, 2016; Landais et al., 2010), whereas some of the higher winter $\Delta^{\prime 17}O$ values may reflect moisture recycling during continental-scale airmass transport (Li et al., 2015; Surma et al., 2021; Tian et al., 2018, 2019). Relative humidity above oceanic moisture sources and atmospheric mixing may be additional drivers of the seasonal $\Delta^{\prime 17}O$ signal (Li et al., 2015; Tian et al., 2018). While we cannot attribute seasonal variation in $\Delta^{\prime 17}O$ in U.S. precipitation to a single process, we use these data to highlight the seasonal pattern of higher winter $\Delta^{\prime 17}O$ values and lower summer $\Delta^{\prime 17}O$ values observed in this study and previous work.

### 5.4. Triple Oxygen Isotope Meteoric Water Line

The triple oxygen isotope meteoric water line was first defined in 2010 from Antarctic snow, Vostok ice (Landais et al., 2008), and a set of surface water, cave water, precipitation, and snow samples collected primarily from Asia and Europe (Luz & Barkan, 2010). This work laid the foundation for more than a decade of research by setting the $\delta^{\prime 18}O-\delta^{\prime 17}O$ regression slope through this sample set (0.528 ± 0.001) as the reference slope and establishing the intercept (0.033 ± 0.003) as the average $\Delta^{\prime 7}O$ value of meteoric water on Earth. Since 2010, these values have provided a point of reference to evaluate isotopic variability and infer information about hydrology, paleoclimate, paleoaltimetry, and the rock cycle (e.g., Bindeman, 2021; Ibarra et al., 2021; Passey & Levin, 2021).

Since 2010 several studies have used water $\Delta^{\prime 17}O$ data to re-evaluate the triple oxygen isotope meteoric water line (Table 1). These re-evaluations are motivated by an understanding that an accurate and representative meteoric water line is critical for applications of $\Delta^{\prime }{}^{17}O$ in both modern and ancient systems and a growing number of meteoric water $\Delta^{\prime 17}O$ data sets. Previously reported regression lines in Table 1 include surface waters that might be evaporated (Aron et al., 2021a; Sharp et al., 2018) or are biased toward polar precipitation (He et al., 2021). By including only precipitation data in this study, we minimize any effects of evaporation and focus on the $\delta^{\prime 18}O-\delta^{\prime 17}O$ relationship from non-polar regions.

Similarities among the slopes and intercepts in Table 1 highlight two important points. First, all of the re-evaluated slopes are less than 0.528, which (a) means that the $\delta^{\prime 18}O$ and $\delta^{\prime 17}O$ values of non-polar precipitation record more than just Rayleigh distillation and (b) sets an expectation that $\Delta^{\prime 17}O$ and $\delta^{\prime 18}O$ values from precipitation and flowing surface water should be slightly anticorrelated (Beverly et al., 2021; Li et al., 2017; Passey & Ji, 2019; Surma et al., 2015, 2018; Voigt et al., 2021). Second, the re-evaluated $\delta^{\prime 18}O-\delta^{\prime 17}O$ relationships of most non-polar waters have intercepts less than 0.033%. Although 33 per meg has been used as a “typical” $\Delta^{\prime 17}O$ value of meteoric water, our results show that this value does not represent either seasonal amount-weighted summer or winter precipitation $\Delta^{\prime 17}O$ in the United States. Future studies should reconsider this assumed $\Delta^{\prime 17}O$ value for meteoric waters in $\Delta^{\prime 17}O$ interpretations and continue to probe the $\delta^{\prime 18}O-\delta^{\prime 17}O$ relationship as it may continue to vary with additional spatial and temporal coverage of samples (Putman et al., 2019).

### 5.5. Utility of $\Delta^{\prime 17}O$ in Paleoclimate Applications and Directions of Future Work

Isotopic techniques to quantify evaporation in modern waters are well established with *d*-excess (e.g., Bowen et al., 2018; Fiorella et al., 2015; Gat, 1996; Tappa et al., 2016; Xia & Winnick, 2021), but the ability to isolate isotopic effects of evaporation has long been a challenge in oxygen isotope paleoclimatology. Reconstructing *d*-excess is challenging for paleoclimate applications because few geologic materials contain both hydrogen and oxygen, with the notable exceptions of fluid inclusions and gypsum where water itself is preserved (e.g., Evans et al., 2018; Wortham et al., 2022). However, given the consistent $\Delta^{\prime 17}O$ response to evaporation, refined estimates of the $\Delta^{\prime 17}O$ values of precipitation make it possible to identify evaporation in oxygen-bearing geologic minerals and improve our understanding of paleoclimate and paleoaltimetry (e.g., Evans et al., 2018; Gázquez et al., 2018; Ibarra et al., 2021; Passey & Levin, 2021).

With additional work, seasonal variations of precipitation $\Delta^{\prime 17}O$ may also add new information to interpret isotopic records. This could be particularly useful for paleoclimate archives that retain isotopic information about climate conditions but are susceptible to isotopic variations related to both seasonality and evaporative enrichment (e.g., Breecker et al., 2009; Kelson et al., 2020). In future hydrologic applications, precipitation $\Delta^{\prime 17}O$ data may shed light on seasonal water use or [CO_2_ uptake by plants (Allen et al., 2019; Hofmann et al., 2017), distinguish water sources in seasonally snow-dominated watersheds (e.g., Jespersen et al., 2018; Tappa et al., 2016), track seasonal variations in evapotranspiration and boundary layer mixing (e.g., Fiorella et al., 2018; Welp et al., 2012), or monitor groundwater or surface water recharge (e.g., Jasechko et al., 2014; Voigt et al., 2021).

Before launching into new directions of paleoclimate triple oxygen isotope research, it is important to study the range and drivers of modern $\Delta^{\prime 17}O$ variability. This is true of all paleoclimate proxies but is especially important for triple oxygen isotopes because $\Delta^{\prime 17}O$ is defined as the deviation from a reference relationship and Table 1 shows that most waters follow a shallower slope and have a lower intercept than the canonical empirical $\delta^{\prime 18}O-\delta^{\prime 17}O$ relationship. This means that for most applications it will be critical to establish local amount-weighted precipitation $\Delta^{\prime 17}O$ values.

Moving forward, additional $\Delta^{\prime 17}O$ data from surface water, water vapor, and precipitation are still needed. Future event-scale and/or integrated monthly precipitation samples collected along elevation, latitudinal, and longitudinal transects will be useful to assess spatiotemporal triple oxygen isotope variability and improve interpretations of $\Delta^{\prime 17}O$ in paleoclimate applications. Surface water samples, which are logistically easier to collect than rain and are often isotopically similar to annual amount-weighted precipitation, will also be useful to explore spatial $\Delta^{\prime 17}O$ patterns and can provide information that is more relevant to the geologic community than individual precipitation samples (e.g., Bershaw et al., 2020).

## 6. Conclusion

This study presents new precipitation $\delta^{18}O$, *d*-excess, and $\Delta^{\prime 17}O$ data from the western and central United States and stream $\delta^{18}O$, *d*-excess, and $\Delta^{\prime 17}O$ data from the Willamette River Basin in western Oregon. The key findings are: (a) precipitation $\delta^{\prime 18}O-\delta^{\prime 17}O$ slopes often differ from the 0.528 reference value, (b) seasonal amount-weighted precipitation $\Delta^{\prime 17}O$ values likely differ for summer and winter, (c) there are different controls on $\Delta^{\prime 17}O$ and $\delta^{18}O$ such that $\Delta^{\prime 17}O$ has the potential to bring additional information, and (d) it is critical to establish the local $\Delta^{\prime 17}O$ variation before using $\Delta^{\prime 17}O$ to characterize evaporation or derive other paleoclimate information.

Putting the $\Delta^{\prime 17}O$ data into context with previous work, the most striking feature of precipitation $\Delta^{\prime 7}O$ variability is the seasonal distinction in $\Delta^{\prime 17}O$ values (higher in the winter, lower in the summer) that is consistent across the globe. These seasonal patterns likely reflect a combination of sub-cloud evaporation, atmospheric mixing, moisture recycling, sublimation, and/or variation in relative humidity at remote moisture sources, along moisture trajectories, and at local collection sites. Additional work is still needed to parse out the fractionating effects of each of these processes on precipitation $\Delta^{\prime 7}O$. Still, it is clear that seasonal variation in $\Delta^{\prime 17}O$ values differs from that of $\delta^{18}O$ and *d*-excess, indicating that $\Delta^{\prime 17}O$ values provide new, complementary information.

Ultimately, controls on precipitation $\Delta^{\prime 7}O$ are complex and comprehensive studies to understand the mechanisms driving its variation should be the focus of future work. Results presented here provide an overview of precipitation $\Delta^{\prime 17}O$ variability, but do not have the spatial or temporal resolution to systematically understand the fractionating process responsible for the observed variation. Future studies with higher temporal and spatial resolution will help investigate synoptic processes responsible for seasonal variation in precipitation $\Delta^{\prime 17}O$ values and to understand spatial variation in $\Delta^{\prime 17}O$. In addition, future studies of water vapor and surface water $\Delta^{\prime 17}O$ will be useful to assess the role of atmospheric mixing, evaluate whether $\Delta^{\prime 17}O$ can be used to identify moisture source regions in North America, and help refine the slope and intercept of the triple oxygen isotope meteoric water line. Although there is still quite a bit left to understand about $\Delta^{\prime 17}O$, initial results are clear that 33 per meg, which is inferred from the intercept of the original triple oxygen isotope line and assumed to represent the average meteoric water $\Delta^{\prime 17}O$ value, can approximate average conditions in some circumstances but might not be appropriate in areas dominated by winter recharge or that have other seasonal dynamics. Future work that refines our understanding of $\Delta^{\prime 17}O$ systematics will improve interpretations of triple oxygen isotope data for paleoclimate, paleoaltimetry, paleoecology, and paleo-atmospheric applications.

## Supplementary Material

Table1

Table2

Supplement1

## Figures and Tables

**Table 1:** Slopes and Intercepts of Meteoric Water $\delta^{\prime 18}O-\delta^{\prime 17}O$ Regression Lines

Reference	Slope	Intercept (%)	Observed or defined	Notes
Meijer and Li (1998) and Barkan and Luz (2005)	0.528	0	Defined	Reference relationship
Luz and Barkan (2010)	0.528	0.033	Observed	All available water data
Sharp et al. (2018)	0.5265	0.014	Observed	All water with $\delta^{18}O$ values > −20%
Aron et al. (2021a)	0.5268	0.015	Observed	All integrated monthly precipitation and flowing rivers
He et al. (2021)	0.5279	0.021	Observed	Tropical, mid-latitude, and polar precipitation and tap water
This study	0.5264	0.014	Observed	Precipitation data only ^a^

a Precipitation data compiled from: Affolter et al. (2015), Aron et al. (2021b), Beverly et al. (2021), Gázquez et al. (2017), Gimenez et al. (2021), He et al. (2021), Landais et al. (2010), Luz and Barkan (2010), Surma et al. (2018), Tian et al. (2019, 2021), and Uechi and Uemura (2019); this study.

## Data Availability

All isotope data from this study are available on the University of Utah Water Isotope Database under Project ID 00388 (Aron et al., 2023), 00011 (Brooks et al., 2012b), and 00016 (Brooks, 2017).

## Key Points:

- Precipitation $\delta^{\prime 18}O-\delta^{\prime 17}O$ slopes often differ from the 0.528 reference value
- Precipitation $\Delta^{\prime 17}O$ values are typically higher in the winter and lower in the summer
- Different controls on $\Delta^{\prime 17}O$ and $\delta^{18}O$ mean that $\Delta^{\prime 17}O$ provides new information for paleoclimate reconstructions
