# Supplementary material for: Seasonal Variations in Triple Oxygen Isotope Ratios of Precipitation in the Western and Central United States
Source: Paleoceanogr Paleoclimatol. Author manuscript; Available in PMC 2024 Mar 13. (PMC10659079; doi:10.1029/2022pa004458)
Supplement: Supplement1 [file NIHMS1939808-supplement-Supplement1.pdf]

Paleoceanography and Paleoclimatology

Supporting Information for

Seasonal variations in triple oxygen isotope ratios of precipitation in the western and central United States

Phoebe Aron [a], Shuning Li [b], J. Renée Brooks [c], Jeffrey M. Welker [d, e, f], Naomi E. Levin [a]

[a] Department of Earth and Environmental Sciences, University of Michigan, Ann Arbor, MI, USA; [b] Institute of Geochemistry, School of Earth and Space Sciences, Peking University, CN; [c] Pacific Ecological Systems Division, Center for Public Health and Environmental Assessment, Office of Research and Development, U.S. Environmental Protection Agency, Corvallis, OR, USA; [d] Department of Biological Sciences, University of Alaska, Anchorage, AK, USA; [e] Ecology and Genetics Research Unit, University of Oulu, Finland; [f] University of the Arctic (UArctic), Rovaniemi, Finland

Contents of this file

Figures S1 to S6
Table S3 to S4

Additional Supporting Information (Files uploaded separately)

Table S1
Table S2

Introduction

This supplement contains Figures S1-S6 and Tables S3-S4 that support the main text.

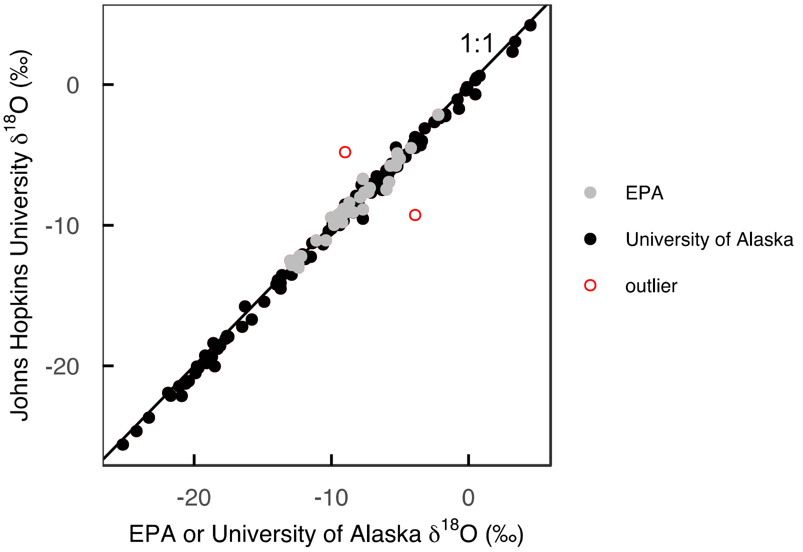

Figure S1. Scatterplot of $\delta^{18}O$ values measured at EPA (solid gray circles) or University of Alaska Anchorage (solid black circles) compared with $\delta^{18}O$ values measured at Johns Hopkins University. $\delta^{18}O$ measurements are identical within analytical precision, except for two outliers that are noted with open red circles. We can find no clear analytical explanation for these outliers. These points are included in Table S1 but are excluded from our analysis.

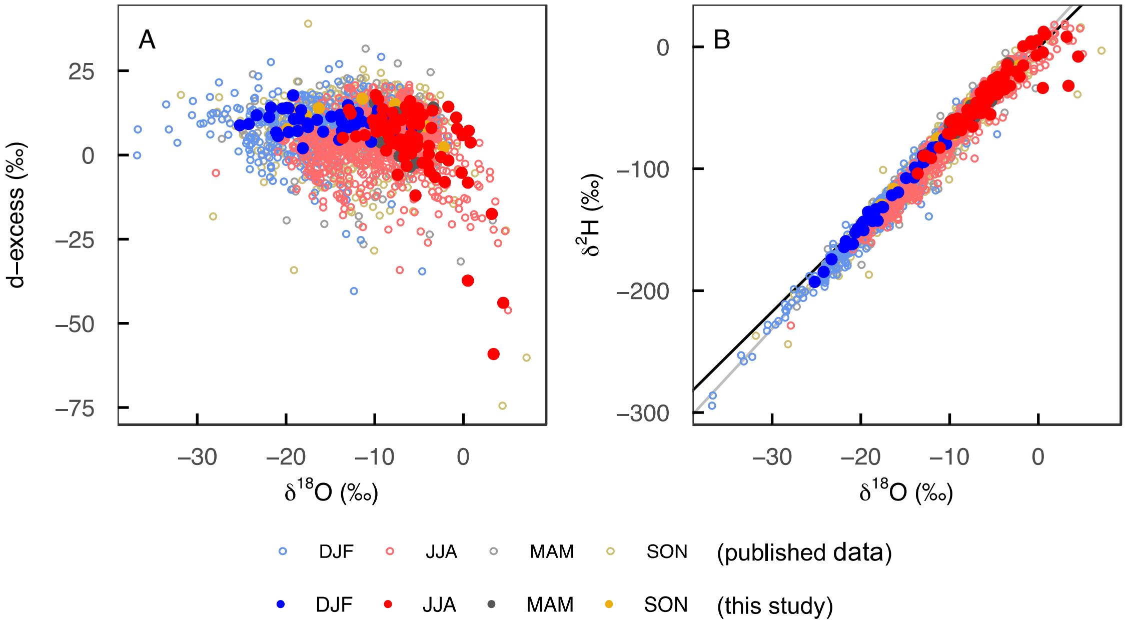

Figure S2. Scatterplots comparing δ^{18}O vs. d-excess (A) and δ^{18}O vs. δ^2H (B) of western and central United States precipitation reported in this study (blue) and published precipitation data from a similar geographic region (gray). The black line in B is the Global Meteoric Water Line. The ranges of δ^{18}O and d-excess values reported in this study are statistically indistinguishable from the previously published data. Published data were accessed from the University of Utah water isotope database (wiDB, waterisotopes.org) in December 2020.

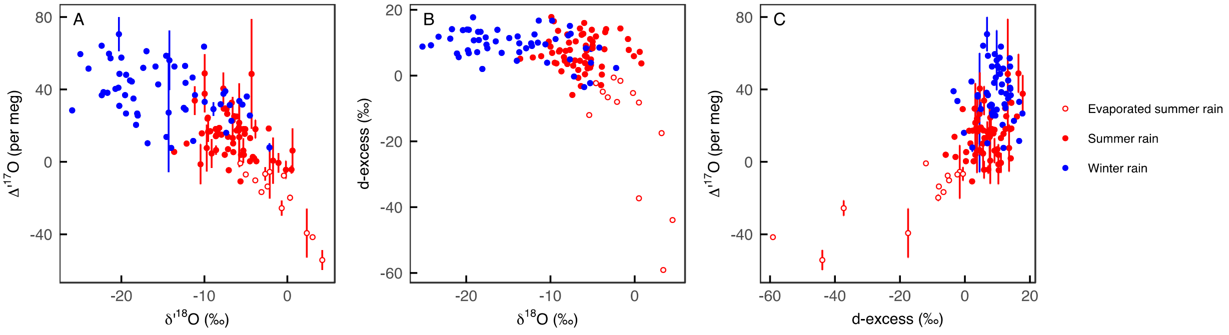

Figure S3. Scatterplots of summer (red) and winter (blue) precipitation δ'^{18}O vs. Δ'^{17}O (A), δ^{18}O vs. d-excess (B), and d-excess vs. Δ'^{17}O (C) from the western and central United States. These are the same data points shown in Figures 4a-4c, but the evaporated summer rains are highlighted with open circles. No winter rains have negative Δ'^{17}O and negative d-excess values.

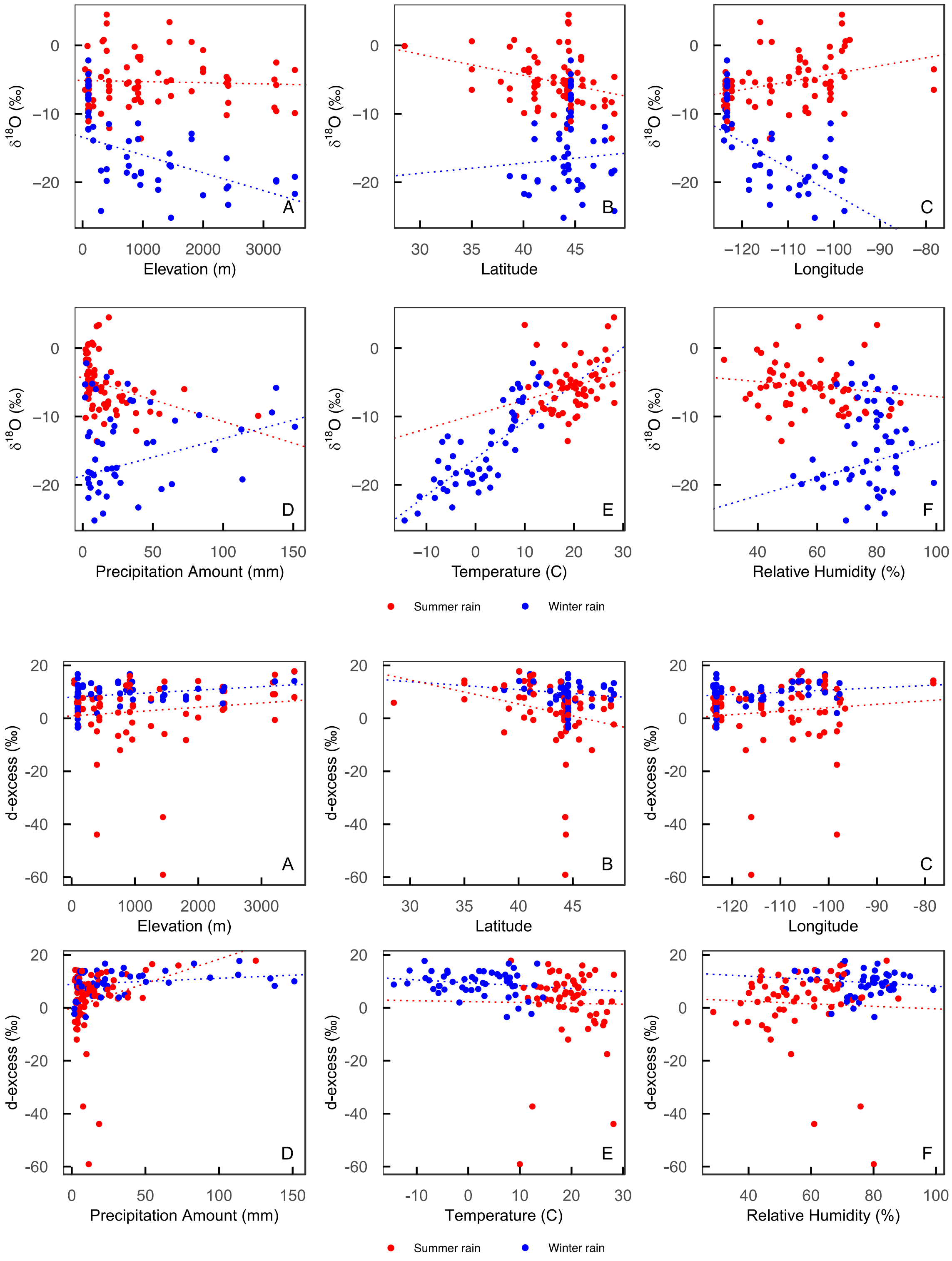

Figure S4. Same as Figure 5 in the main text but showing $\delta^{18}O$ and d-excess.

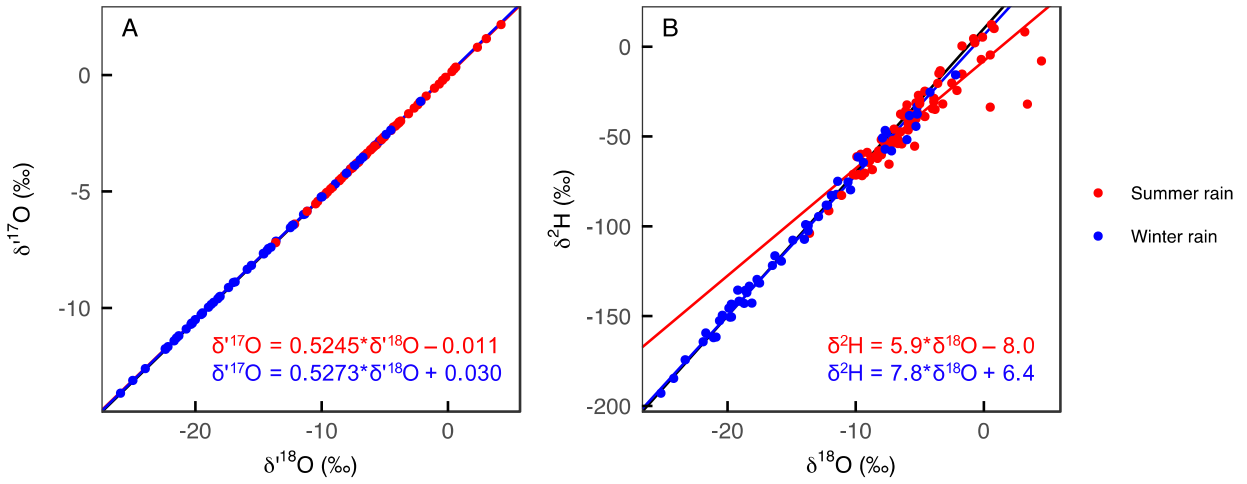

Figure S5. Same as Figure 3 in the main text but showing only precipitation data and differentiating summer (red) and winter (blue) samples.

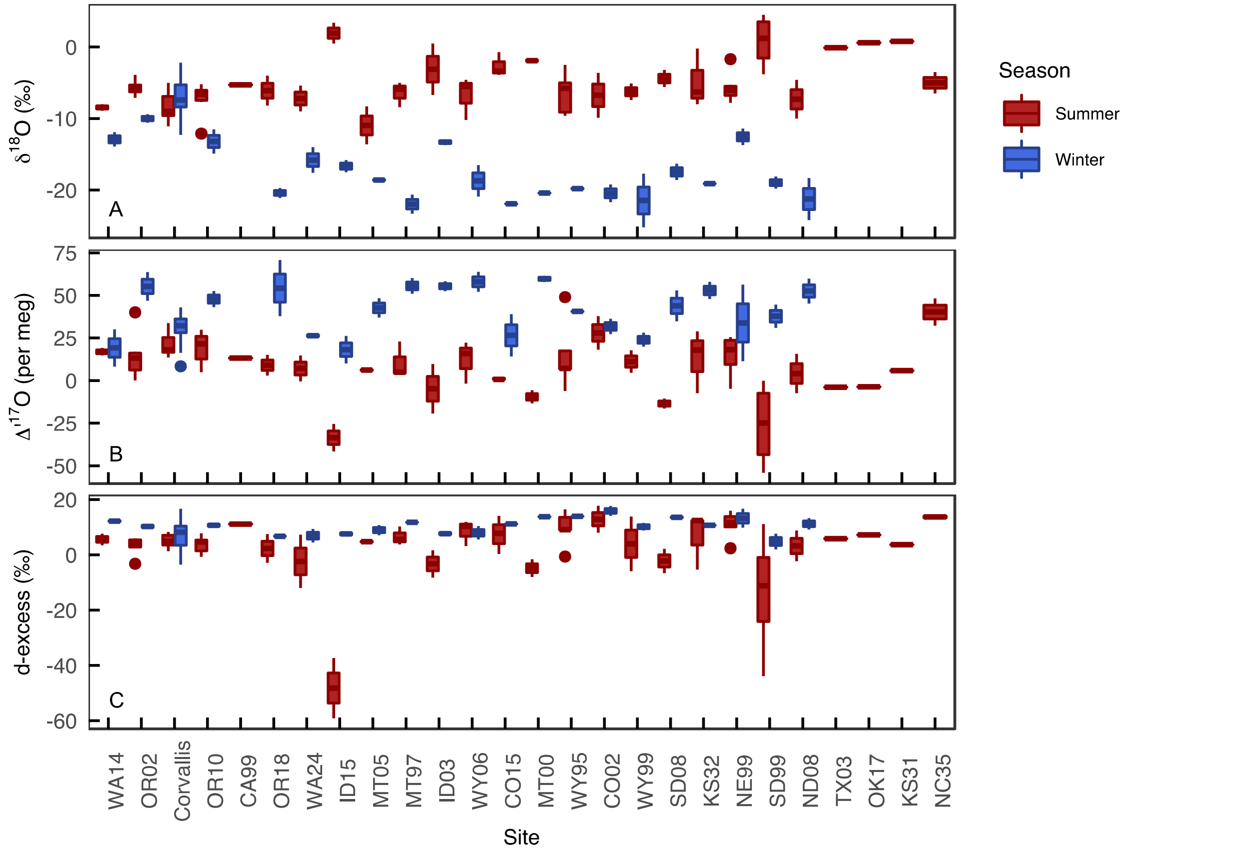

Figure S6. Box plots of summer (red) and winter (blue) precipitation $\delta^{18}O$ (A), $\Delta'^{17}O$ (B), and d-excess (C). Sites are listed longitudinally with western-most sites (Washington and Oregon) on the left and the eastern-most site (North Carolina) on the right. Same as Figure 6 in the main text, but with all the precipitation data.

Table S1. Sample and average isotopic data reported in this study. Note that $\delta^{18}O$ and $\delta^{2}H$ data in Table S1 were originally reported in Welker, 2012 or Brooks et al., 2010. The reported average $\delta'^{18}O$ and $\delta'^{17}O$ values were measured at Johns Hopkins University as part of this study.

Table S2. Raw isotopic data reported in this study.

Table S3. Reported average ± standard deviation (per meg) $\Delta'^{17}O$ values of USGS reference waters.

Sample ID	This Study	Aron et al., 2021	Berman et al., 2013
USGS45	20 ± 16	13 ± 7	12 ± 1
USGS46	25 ± 7	19 ± 11	20 ± 2
USGS47	40 ± 8	32 ± 9	40 ± 1
USGS48	30 ± 10	31 ± 6	26 ± 3

Table S4. Previously observed precipitation $\delta'^{18}O$–$\delta'^{17}O$ slopes.

Reference	Location	$\delta'^{18}O$–$\delta'^{17}O$ Slope	Sample Collection Timeframe
Tian et al., 2021	Western Namibia	0.5184	Sporadic
Gimenez et al., 2021	Northern Spain	0.524	Per event
Beverly et al., 2021	Northern Tanzania	0.5250	Per event
Landais et al., 2010	Southwestern Niger	0.5257	Per event
Affolter et al., 2015	Northwestern Switzerland	0.5269	Annual
He et al., 2021	Singapore	0.5271	Monthly
Aron et al., 2021b	Southern Peru	0.5275	Bi-weekly
Tian and Wang, 2019	Central United States	0. 5275	Per event
Gázquez et al., 2017	Southeastern Spain	0.5282	Per event
Uechi and Uemura, 2019	Southern Japan	0.5296	Weekly
